# Genome-Wide Identification, Characterization, and Transcriptional Profile of the HECT E3 Ubiquitin Ligase Gene Family in the Hard-Shelled Mussel *Mytilus coruscus* Gould

**DOI:** 10.3390/genes15081085

**Published:** 2024-08-16

**Authors:** Feng Guo, Zhenqi Xin, Zhenyu Dong, Yingying Ye

**Affiliations:** 1 National Engineering Research Center for Marine Aquaculture, Zhejiang Ocean University, Zhoushan 316022, China; agsguo@163.com; 2 Marine Science and Technology College, Zhejiang Ocean University, Zhoushan 316022, China; darkxin19991015@163.com (Z.X.); dongzhenyu2021@163.com (Z.D.)

**Keywords:** *Mytilus coruscus*, HECT gene family, expression profiling, bioinformatics, biotic and abiotic stress

## Abstract

The homologous E6-AP carboxy-terminal structural domain (HECT) contained in E3 ubiquitin ligases (E3s) is a key factor in protein degradation and maintenance of cellular homeostasis in animals. However, the functional roles and evolutionary aspects of the HECT gene family in bivalve mussels remain unclear and warrant further investigation. In this study, we identified 22 HECT genes within the genome of *Mytilus coruscus* Gould, all containing a conserved HECT structural domain derived from dispersed repeats, distributed unevenly across 11 chromosomes. Phylogenetic analysis classified *M. coruscus* HECT genes into six major classes, with amino acid sequences within the same evolutionary clade displaying similar conserved motifs. Homology analysis with HECT genes of four bivalve species revealed that *M. coruscus* and *Mytilus galloprovincialis* possessed the largest number of homologous gene pairs, showing a significant correlation between the two in the evolution of the HECT gene family. Homology analysis with HECT genes of four bivalve species revealed that *M. coruscus* and *M. galloprovincialis* possessed the largest number of homologous gene pairs, showing a significant correlation between the two in the evolution of the HECT gene family. *M. coruscus* exhibited pronounced and specific expression in gills and blood tissues. Notably, *Mco_UPL3* gene expression was significantly upregulated after 12 h of acute heat stress (33 °C) and 24 h of *Vibrio* injection (0.4 OD). Gene ontology analysis of the HECT genes in *M. coruscus* revealed that it is primarily enriched in protein modification and degradation functions. This suggests that HECT genes may play a key role in protein degradation and immunomodulation in *M. coruscus*. These findings offer valuable insights for the breeding of stress-tolerant traits in *M. coruscus*. In summary, our data shed light on the potential functions of HECT E3 ligases in response to heat stress and *Vibrio* infection, providing practical guidance for enhancing resilience through breeding in *M. coruscus*.

## 1. Introduction

Ubiquitination represents a fundamental biological process highly prevalent in eukaryotes. It encompasses a diverse array of essential functions, encompassing not only its well-established regulatory role at the protein level but also precise and ancillary functions in endocytosis, cellular signaling, DNA repair, and the regulation of gene expression [1,2,3]. Upon the binding of ubiquitin molecules to specific substrate proteins, the ubiquitinated proteins undergo degradation orchestrated by the 26S proteasome complex. These intricate processes involve a three-step enzymatic reaction catalyzed by ubiquitin-activating enzyme (E1), ubiquitin-conjugating enzyme (E2), and ubiquitin ligase (E3). Specifically, the activation of the ubiquitin molecule by E1, facilitated by ATP, precedes its transfer to E2. Subsequently, the ubiquitin ligase E3 binds and facilitates the transfer of the ubiquitin moiety to its substrate, culminating in the ATP-dependent degradation of the 26S proteasome [4,5,6]. Notably, the E3 ubiquitin ligase assumes a pivotal role in determining the ubiquitination and subsequent degradation of the substrate. Given the direct binding capability of E3 ligases to substrates and their role in specifying the ubiquitin system’s specificity, a substantial number of E3 ligases exist across various organisms, in stark contrast to the relatively limited number of E1 and E2 ligases [1,7]. Classifying E3 ligases based on their structure and function reveals four main types: HECT-type, U-box-type, RING-finger-type, and RBR-type. Intriguingly, these diverse E3 ligase types exhibit low sequence homology, with significant differences in their compositions [7].

The HECT (homologous to E6AP carboxy-terminal) E3 ligase family stands as one of the earliest and extensively studied E3 ligase families [2]. Based on the N-terminal structural domain, HECT E3 ligases can be categorized into three groups: the Nedd4 family (9 members), the HERC family (6 members), and another HECT family (13 members) [1]. Alongside the shared HECT C-terminal domain, the Nedd4 subfamily distinguishes itself with the presence of the WW and C2 domains, which have been subjects of intensive investigation. The C2 structural domain at the N-terminal end exhibits an ability to bind Ca^2+^ and phospholipids. This feature is crucial not only for directing proteins to phospholipid membranes but also for contributing to the ubiquitination of targeted substrate proteins [8,9,10].

The HERC subfamily is characterized by the inclusion of one or more rcc-like domains (RLDs) [11]. Depending on the number of RLDs, the HERC subfamily further divides into two large HERCs and four small HERCs. Additionally, several other HECT ligases, such as E6AP and HUWEI, exist. E6AP, the inaugural member of the family, incorporates a zinc-binding folding structural domain, AZUL (the Zn-finger structural domain of the amino-terminus of Ube3a ligases). Conversely, HUWE1 features a WWE structural domain and a ubiquitin-associated (UBA) structural domain, with a primary focus on cancer development [12,13].

The HECT E3 ubiquitin ligases play pivotal roles in animals, actively participating in processes such as protein degradation and the maintenance of cellular homeostasis [13,14,15,16,17]. Exploration of the evolutionary patterns of HECT E3 ubiquitin ligases in animals has enriched our comprehension of cellular and protein regulatory mechanisms. Moreover, it has been evidenced that animals employ the ubiquitin–proteasome system as a selective mechanism for degrading specific proteins, acting as an adaptive response to environmental stresses [18,19,20,21,22]. This system effectively governs intracellular signaling and metabolic processes by selectively degrading proteins. Particularly in aquatic animals, this regulatory mechanism assumes significance in coping with stresses such as temperature changes and microbial infections [23].

Significant strides have been achieved in ubiquitination investigations within the realm of aquaculture animals, particularly in fish, with a notable focus on antiviral immune modulation. Notably, TRIM (tripartite motif) family proteins, acting as E3 ubiquitin ligases, have emerged as pivotal players in the innate antiviral immune response in crucian carp [24]. The E3 ubiquitin ligase ring finger protein 114 (RNF114) in sea perch (*Lateolabrax japonicus*) [25] acts as an inhibitor of the RLR signaling pathway during infection with red grouper neuron necrosis virus (RGNNV) [26]. In the domain of shellfish research, studies encompassing Philippine clams (*Ruditapes philippinarum*) [23], hard-shelled mussels (*M. coruscus*) [18], Pacific oysters (*Crassostrea gigas*) [27], and abalone (*Haliotis Genus*) [28] under heat stress have unveiled the significant mediation of relevant gene expression by ubiquitin. Furthermore, ubiquitination significantly influences homeostatic repair mechanisms in vivo through signal transduction and gene regulation when *Artemia franciscana* is subjected to salt stress [29]. Lin et al. elucidated the crucial role of the HECT gene in immune regulation under Vibrio eel infection by identifying and analyzing the HECT gene in Philippine clams [23]. Additionally, the study conducted by Song et al. demonstrated that CgWWP1, functioning as a ubiquitin protein ligase, actively participates in the regulation of granulocyte proliferation.

The hard-shelled mussel (*M. coruscus* Gould), classified under the phylum Mollusca (Mollusca), the class Bivalvia (Bivalvia), the order Mytilida (Mytilidae), the family Mytilidae, and the genus *Mytilus*, primarily inhabit temperate waters in East Asia, including China’s East China Sea, the Yellow Sea, and the Bohai Sea. The primary concentration of aquaculture activities for *M. coruscus* is observed in Shengsi County, Zhoushan City, Zhejiang Province. Recognized as one of the economically valuable shellfish in marine aquaculture in China [30,31,32,33], understanding the mechanisms underlying intertidal shellfish adaptation to elevated temperatures has garnered significant attention in aquatic biology research. This interest arises from the escalating summer temperatures, the heightened frequency of extreme heat waves, and the observed collective mortality events in species such as mussels and barnacles caused by high temperatures [34,35,36,37]. Notably, bivalve shellfish show ubiquitin binding as an indicator of heat damage, emphasizing the significant effect of heat exposure on ubiquitinated protein levels [38]. In addition, the problem of diseases caused by Vibrio-like bacterial stress during bivalve production has become a major bottleneck affecting the industry [39].

To date, the sequence characterization and function of HECT have been elucidated in *R. philippinarum* [23]. However, in the context of bivalve mussels, the functionality and evolutionary aspects of the HECT gene family remain relatively obscure, demanding immediate and thorough investigation. In this study, we identified and characterized the HECT genes from the genome of *M. coruscus*. Our investigation encompassed a comprehensive analysis of gene structures and chromosomal locations of HECT, along with an exploration of the evolutionary relationships among different bivalve species. This analysis involved the construction of phylogenetic trees, examination of motif composition, and covariance analysis. Furthermore, we delved into the expression patterns of HECT genes in *M. coruscus* under both abiotic stress (high-temperature stress) and biotic stress (Vibrio stress). The meticulous examination of the *M. coruscus* genome and its transcriptome furnishes crucial insights for analyzing the function and evolution of the mussel HECT gene family.

## 2. Materials and Methods

### 2.1. Identification of HECTs in M. coruscus

The chromosomal-level genome of *M. coruscus* was derived from proprietary data annotated by our laboratory assembly (unpublished data). We obtained the Hidden Markov model (HMM) profile for the HECT domain (PF00632) from the Pfam protein family database (http://pfam.xfam.org) (accessed on 7 March 2024) [40]. Utilizing HMMER software (v3.0) with default parameters [41], we extracted protein sequences of the HECT gene family from the genomic data of *M. coruscus*. Following the manual elimination of redundant sequences, we validated the conserved HECT domains of candidate proteins through scrutiny with the Conservative Domain Databases (CDD) (https://www.ncbi.nlm.nih.gov/Structure/cdd/wrpsb.cgi) (accessed on 12 March 2024) [42], SMART (http://smart.embl.de/) (accessed on 13 March 2024) [43], and the PANTHER classification system (http://pantherdb.org/) (accessed on 13 March 2024) [44]. Protein sequences lacking the HECT domain were subsequently excluded. Additionally, we predicted key physicochemical properties of the HECT protein, including amino acid count, theoretical molecular weight (kDa), and isoelectric point (pI), employing calculations through the online program ExPASyProtParam (https://web.expasy.org/protparam/) (accessed on 18 March 2024) [45].

### 2.2. Bioinformatics Analysis of HECT Genes

Utilizing the genome annotation file, the TBtools tool (https://www.tbtools.com/) (accessed on 1 April 2024) [46] was employed to analyze the chromosomal location and gene structure of HECT genes in *M. coruscus*. Furthermore, TBtools was employed for the generation of diagrams illustrating the motif, chromosomal location, and gene structure. The identification of PFAM domains was conducted using the SMART program. CDD was employed for the prediction of conserved domains in HECTs (E-value < 0.001), followed by protein structure visualization using DOG 2.0 software (http://dog.biocuckoo.org) (accessed on 5 April 2024) with default parameters [47]. Multiple Expectation Maximization for Motif Elicitation (MEME) software (v5.5.3) (https://meme-suite.org/meme/) (accessed on 5 April 2024) [48] was employed to scrutinize conserved motifs within HECT sequences. This investigation incorporated the following parameters: the maximum number of motifs set to 10, and the preferred motif width spanning from 6 to 50 amino acid residues.

### 2.3. Construction of Phylogenetic Tree

The construction of the phylogenetic tree relied on HECT gene sequences identified from *M. coruscus* and various other selected species. Representative model organisms encompassed nematode (*Caenorhabditis elegans*), fruit fly (*Drosophila melanogaster*), zebrafish (*Danio rerio*), and humans (*Homo sapiens*). Within the Mytilidae family, *M. coruscus* and *M. galloprovincialis* stood out as notable species. Other bivalve species included *R. philippinarum*, *Patinopecten yessoensis*, and *C. gigas* (Table S1). Protein sequences for these species were obtained from the NCBI database, the UniProt database [49] (https://www.uniprot.org/) (accessed on 13 April 2024), MolluscDB [50] (http://mgbase.qnlm.ac/home) (accessed on 13 April 2024), and previously published articles (Table S2). Subsequently, the protein sequences were named based on their respective entries in the genome file. Multiple protein sequences underwent alignment using the MUSCLE technique with default parameters. The results were subjected to analysis utilizing the JTT matrix model, and a maximum likelihood (ML) phylogenetic tree was constructed through MEGAX (version 10.2) [51] with 1000 bootstrap replicates. Finally, the Evolview website (https://evolgenius.info/) (accessed on 25 April 2024) [52] was employed for the classification and visualization of the phylogenetic tree.

### 2.4. Collinearity Analysis

Gene duplication identification between *M. coruscus* and other selected bivalves was executed using the MCScanX program [53]. In this procedure, the DIAMOND software (v2.1.9) [54] was employed with specific parameters: a maxtarget-seqs set to 5 and an E-value threshold of 8 × 10^−10^. To achieve this, bidirectional BLASTP sequencing was conducted using whole-genome protein sequences of *M. coruscus* against those of *M. galloprovincialis*, *M. yessoensis*, and *C. gigas*. The outcomes of the BLASTP analysis served as input data for the MCScanX software (v0.8), alongside GFF files. Subsequently, the results obtained were visualized and synthesized under the guidance of JCVI.

### 2.5. Subcellular Localization and Protein Structure Prediction

We conducted subcellular localization analysis using WoLF PSORT (https://wolfpsort.hgc.jp/) (accessed on 27 April 2024) [55]. Protein secondary structure prediction employed SOPMA (http://npsa-pbil.ibcp.fr/cgi-bin/npsa_automat.pl?page=npsa_sopma.html) (accessed on 29 April 2024) [56]. To identify protein models with homology to the HECT gene, we queried the PDB database (http://www.rcsb.org/) (accessed on 29 April 2024) [57]. Subsequently, we predicted tertiary structures of the proteins through homology modeling, utilizing the default parameters of the Swiss Model (https://www.swissmodel.expasy.org/) (accessed on 30 April 2024) [58]. The quality of the resulting models was assessed using SAVES v.6.0 (http://servicesn.mbi.ucla.edu/SAVES/) (accessed on 1 May 2024) [59].

### 2.6. Characterization of HECT Gene Expression

All RNA-seq data used in this study are fully summarized in the Supplementary file (Table S3). Our dataset covers RNA-seq data collected from five different organs or tissues of *M. coruscus*: blood, gill, mantle, gonad, and foot. In addition, transcriptome data were obtained from species subjected to abiotic (temperature) and biotic (*Vibrio alginolyticus*) stresses. We scrutinized the expression profile of the HECT gene using these different data sets.

Adult *M. coruscus* were collected from Shengsi Island, Zhoushan City, Zhejiang Province, China, and acclimated in the laboratory at 18 °C for one week. Before the experiment, the mussels were starved for 12 h and then randomly assigned to two groups in a temperature-controlled incubator set at 18 °C. Each group was placed in three 16 L temperature-controlled incubators, with nine mussels per incubator. The experimental group was exposed to high-temperature stress at 33 °C, while the control group was maintained at 18 °C. Samples were collected from both groups at 0 and 12 h, with nine mussels from each incubator per group at each time point, totaling 18 samples. Collected gill, mantle, and adductor muscle tissues underwent RNA extraction for subsequent transcriptome analysis.

For biotic stress, adult *M. coruscus* were collected from Gao Yun Market in Zhoushan City, Zhejiang Province, China, and temporarily stored in a temperature-controlled incubator at 20 °C, where they were acclimated for 7 days. After acclimation, 20 mussels were selected for infection with *V. alginolyticus*. The bacteria were cultured in LB medium at 37 °C for 4 h until the OD600 reached 0.4–0.6. The culture was then centrifuged, and the resulting pellet was resuspended in sterile PBS. The bacterial concentration was adjusted to an OD600 of 0.4, and 100 μL of the suspension was injected into the adductor muscle of each mussel. The remaining 10 mussels, which were not injected, served as controls. Twenty-four hours post-injection, three mussels from each group were randomly selected for dissection, and tissues from the adductor muscle, gills, and mantle were collected for transcriptome sequencing.

Additionally, all HECT genes were functionally annotated and analyzed through gene ontology (GO) using the online database eggNOG-mapper (http://eggnog-mapper.embl.de/) (accessed on 8 August 2024) [60], with the aim of classifying these genes for functional enrichment based on the annotation results.

## 3. Results

### 3.1. Identification and Characterization of HECT Gene Family Members in Mussel

In this investigation, a total of 22 HECT family members homologous to *M. coruscus* were identified. Table 1 provides comprehensive information for all members of the HECT gene family, including details such as name, ID, chromosomal location, chromosome length, intron count, protein length (aa), HECT domain, theoretical PI, and protein molecular weight (kDa). Notably, within this group, *Mco_UPL2* featured the shortest conserved structural domain, encompassing 75 amino acids, while *Mco_HECTD1* exhibited the longest conserved structural domain, spanning 507 amino acids. The theoretical PI and molecular weight of the HECT proteins ranged from 4.6 to 9.29 and 15,283.79 to 427,315.87 kDa, respectively. These 22 members of the HECT gene family are distributed across 11 chromosomes, with chromosome 1 hosting the highest number, containing four family members.

### 3.2. Phylogenetic Analysis of the HECT Gene Family among Species

**Figure 1:**
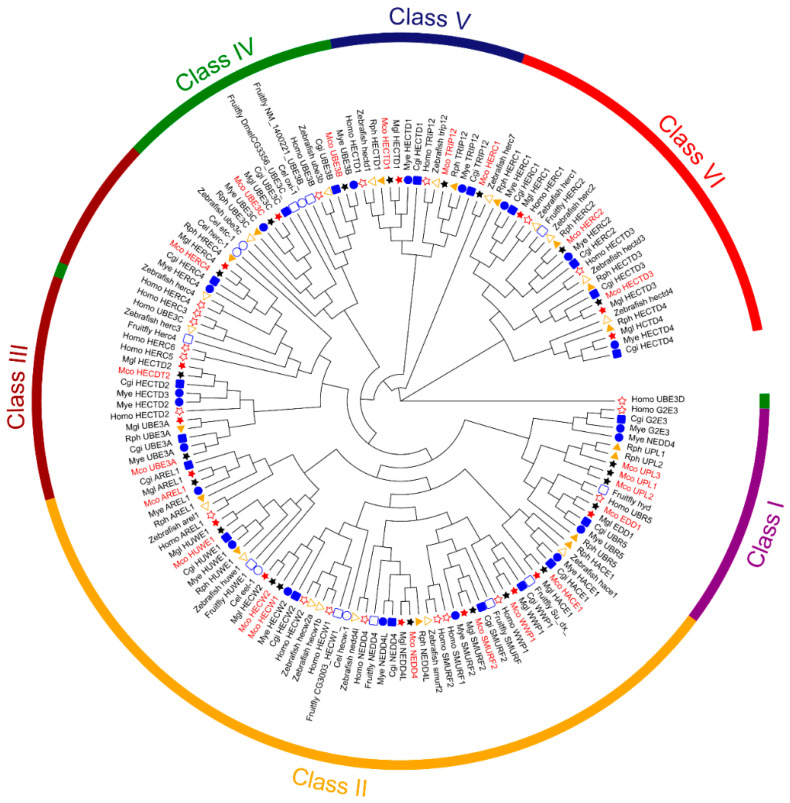
Phylogenetic maximum likelihood (ML) tree analysis of HECT family members of *M. coruscus*. The HECT gene family is subdivided into six categories represented by six colors, with different colored symbols representing different species.

We utilized the MEGA tool to generate a Maximum Likelihood (ML) tree for the analysis of interrelationships among HECT genes. A total of 148 amino acid sequences from nine different species were compared. The HECT genes of these species were annotated using genomes from the NCBI database, protein sequences from the mentioned research paper (*R. philippinarum*) [23], and our unpublished laboratory genome files (*M. coruscus* and *M. galloprovincialis*). The protein sequences are designated according to the branches of the HECT gene family phylogenetic tree and are distinguished by different colors and shapes (Figure 1). We identified six major groups of proteins with different structural domains based on prior studies [5], which include IQ structural domains (class IV), armadillo sequences (class V), EDD structural domains (class I), NEDD4 subfamily structural domains (class II), and HERC subfamily structural domains (class VI). The NEDD4 subfamily significantly contributes to the breadth and size of the HECT gene family. Our investigation revealed 22 HECT sequences in the *M. coruscus* genome distributed across 15 subfamilies (Table 2). Notably, the UPL1-3, UBE3B/3C, and LARGE HERCs subfamilies contain three, two, and two sequences, respectively, in the *M. coruscus* genome. The NEDD4 subfamily harbors five sequences in the *M. coruscus* genome, while four sequences are found in other bivalves, with only one sequence in *R. philippinarum*. It is noteworthy that G2E3 or HECTD4 is absent in *M. coruscus*, and the remaining subfamilies in *M. coruscus* have only one sequence each.

### 3.3. Analysis of Conserved Domains, Motif Discovery, and Gene Structures

**Figure 2:**
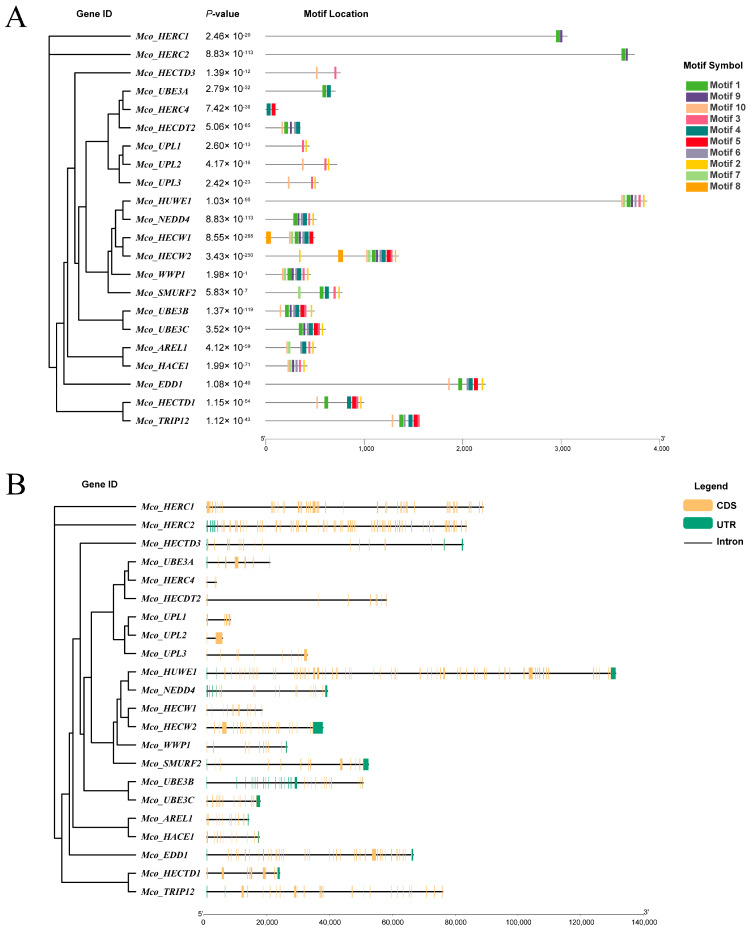
Conserved motifs and gene structure of HECT in *M. coruscus* based on phylogenetic relationships. (**A**) Phylogenetic analysis (**left**) and motif analysis (**right**) of the HECT genes in *M. coruscus*, (**B**) gene structure analysis.

The analysis revealed variability in motif numbers within the HECT gene family in the *M. coruscus* genome, ranging from 2 to 10 motifs. Notably, sequences such as *Mco_HERC1*, *Mco_HERC2*, *Mco_HECTD3*, *Mco_UBE3A*, *Mco_HERC4*, *Mco_UPL1*, *Mco_UPL2*, and *Mco_UPL3* exhibited a limited count of 1 to 3 motifs, sharing similarities with motifs found in other sequences. Duplicated homologs, such as *Mco_HECW* and *Mco_UPL*, displayed comparable motif arrangement structures within their protein configurations (Figure 2A).

To understand the evolutionary conservation of this gene family, we scrutinized the gene structure of HECT genes using *M. coruscus* genome annotation files (Figure 2B). The disparity in coding sequences (CDS) versus intron structures among these HECT genes suggests potential diversity in their biological functions. Results indicated a spectrum of CDS counts ranging from 3 to 58, with approximately one-third of the sequences showing over 20 CDS counts. Notably, *Mco_HERC1*, *Mco_HERC4*, *Mco_HECDT2*, *Mco_UPL1*, *Mco_UPL2*, *Mco_UPL3*, and *Mco_TRIP12* lacked untranslated regions (UTR).

**Figure 3:**
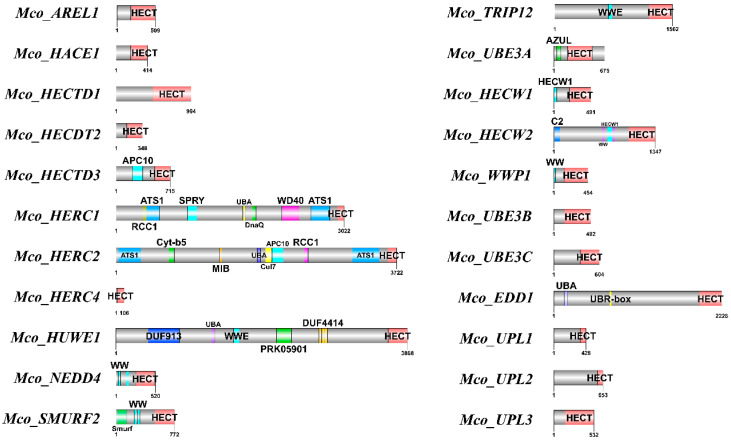
Twenty-two sequences of HECT proteins with predicted conserved structural domains. The gray bar indicates the length of each protein sequence, and the conserved domains are indicated by the pink boxes, while the other colors indicate other conserved domains.

Further characterization of the HECT proteins’ structures was conducted using the NCBI Conservative Domain Database (https://www.ncbi.nlm.nih.gov/cdd/) (accessed on 3 May 2024). The investigation identified 26 proteins in *M. coruscus* potentially containing HECT structural domains (Figure 3). These structural domains ranged in length from 23 to 507 amino acids and included significant domains such as the WW domain, SPRY domain, ATS1 domain, C2_Smurf-like domain, UBR-box domain, DUF domain, HECW1_helix domain, and UBA_HERC2 domain.

### 3.4. Chromosomal Localization of the HECT Gene Family

**Figure 4:**
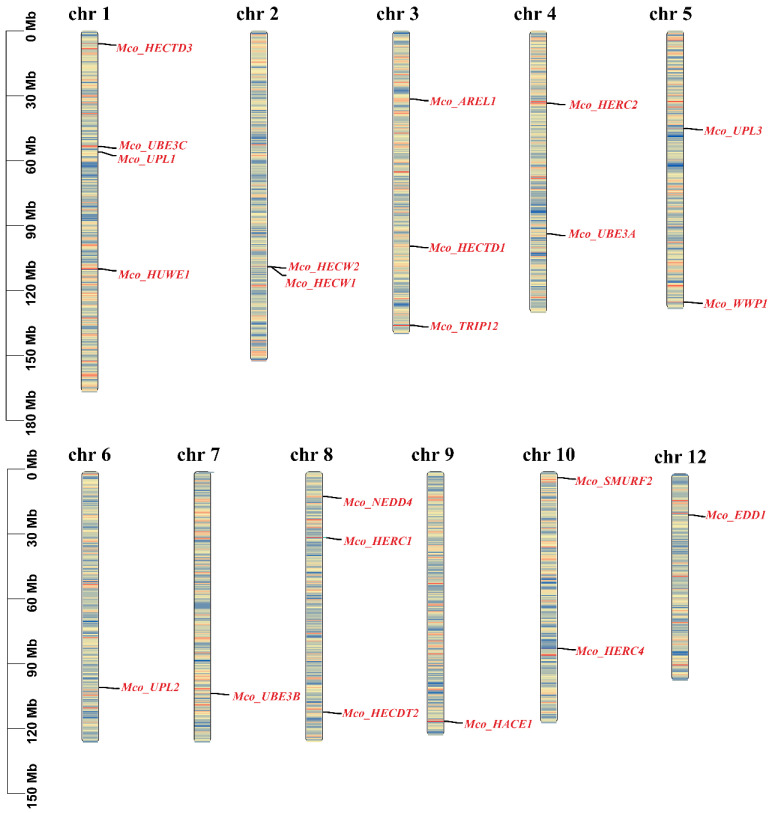
Chromosomal localization and gene density of the HECT genes in *M. coruscus.* The red color on the chromosome represents high-density regions, the blue indicates low-density regions, and the black line marks the location of the gene on the chromosome.

The TBtools software (v2.096) was employed to visualize the chromosomal distribution of HECT genes in the *M. coruscus* genome, revealing an uneven distribution of 22 HECT genes across 11 out of the 14 chromosomes (Figure 4 and Table 1). Duplicated HECT genes were observed on distinct scaffolds, with *Mco_UPL1*, *Mco_UPL2*, and *Mco_UPL3* situated on chromosome 1, chromosome 6, and chromosome 5, respectively.

### 3.5. Secondary Homology Modeling of HECT Protein Structures and Subcellular Localization

**Figure 5:**
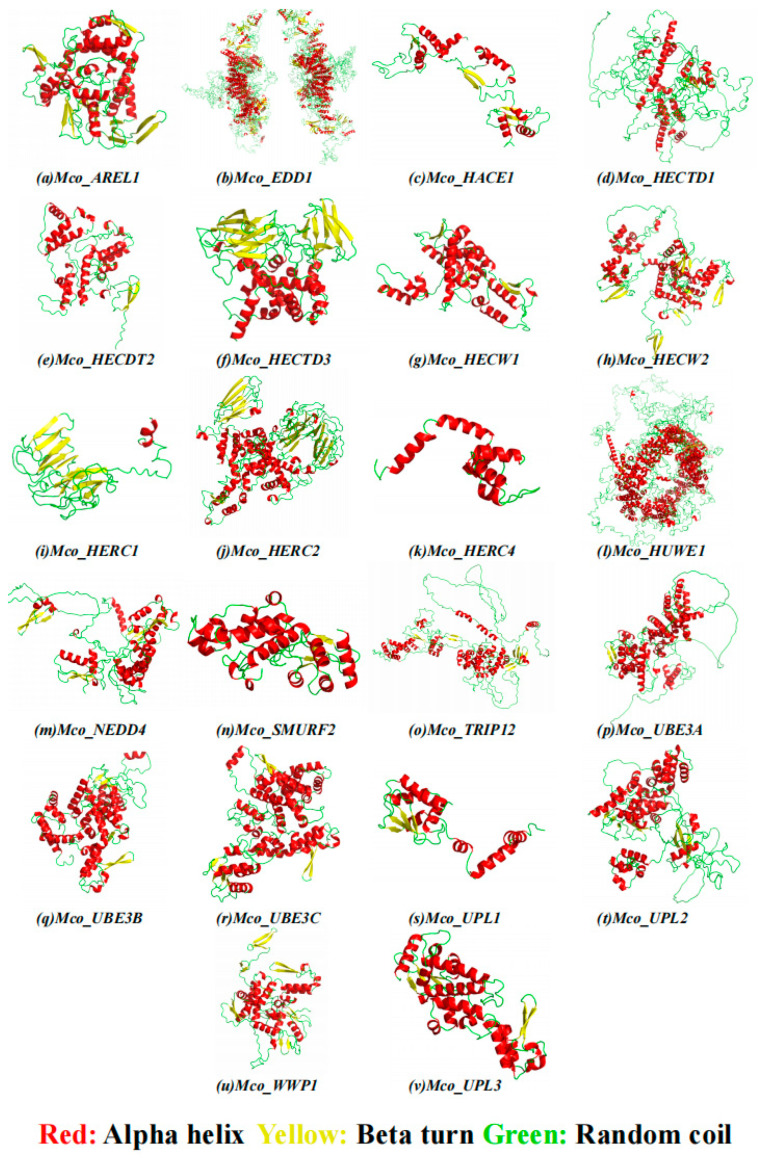
Three-dimensional structure of 22 HECT proteins from *M. coruscus*. Secondary structure elements include α-helices (red), β-turns (yellow) and random coils (green).

The *M. coruscus* HECT gene family exhibits diverse subcellular localizations encompassing the nucleus, cytoplasm, plasma membrane, and mitochondria (Table 3). Regarding the secondary structure of the 22 encoded proteins by *M. coruscus* HECT genes, they predominantly consist of alpha helices and random helices. Specifically, alpha helices ranged from 28.54% to 53.54%, beta turns from 4.10% to 7.49%, random helices from 28.88% to 51.22%, and elongated chains from 8.79% to 21.16% (Table 3 and Figure 5). Notably, proteins from distinct groups within the same species exhibited substantial differences, highlighting the structural diversity prevalent within the HECT family.

### 3.6. HECT Gene Colinearity Analysis

**Figure 6:**
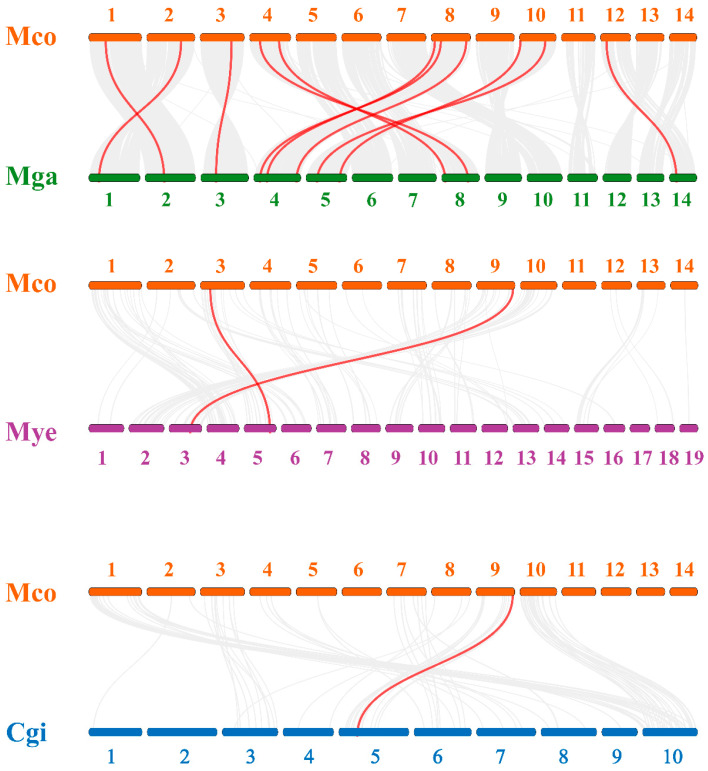
Covariance analysis of *M. coruscus* HECT genes with *M. galloprovincialis* (Mga), *M. yessoensis* (Mye), and *C. gigas* (Cgi). Gray lines in the background indicate colinear blocks in the genomes of *M. coruscus* and other bivalves, while red lines highlight colinear HECT gene pairs.

To thoroughly investigate the phylogenetic mechanisms within bivalve HECT genes, we examined the homology of HECT genes across four bivalve species (*M. coruscus*, *M. galloprovincialis*, *M. yessoensis*, and *C. gigas*) (Figure 6). The HECT genes of *M. coruscus* exhibited homology with those of the selected bivalve species, demonstrating conserved homologous relationships in *M. galloprovincialis* (11 homologous pairs of genes across Chr1, Chr2, Chr3, Chr4, Chr5, Chr8, and Chr14), *M. yessoensis* (2 homologous gene pairs on Chr3 and Chr5), and *C. gigas* (1 homologous gene pair on Chr5) chromosomes, as depicted in Figure 6. In the analysis of HECT gene homology between *M. coruscus* and *M. yessoensis*, we identified *AREL1* and *HACE1* associated with a homologous gene pair. Additionally, in the covariance analysis of HECT genes between *M. coruscus* and *C. gigas*, HACE1 was linked with a covariant gene pair. These findings suggest the potential pivotal role of HACE1 in the evolutionary trajectory of the HECT family among bivalve species.

### 3.7. Analysis of HECT Gene Expression Patterns

**Figure 7:**
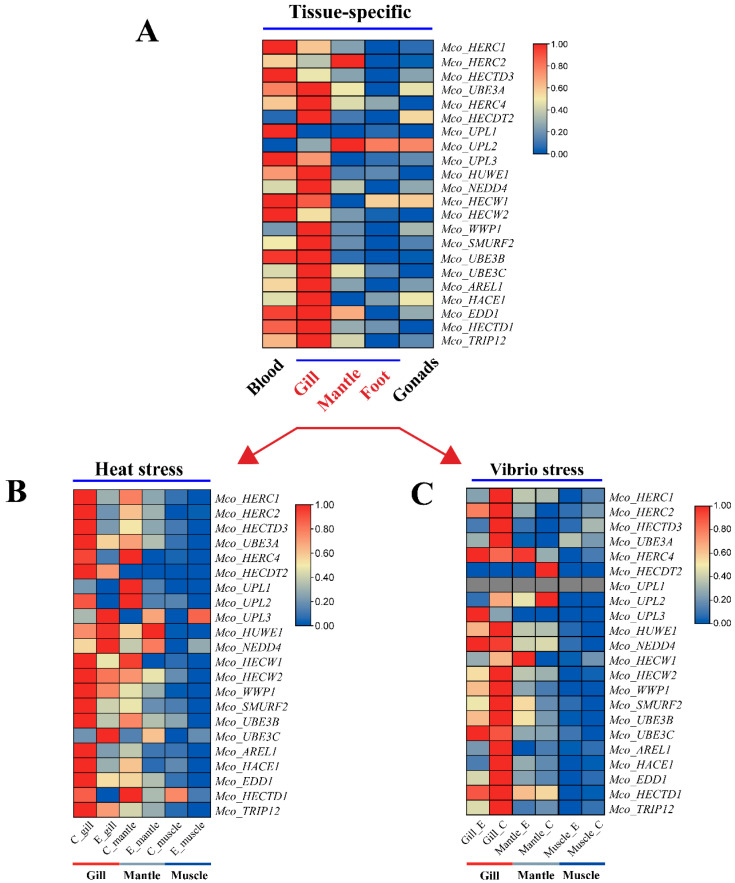
Heat map of the expression of HECT family members in different tissues of adult *M. coruscus* under abiotic and biotic stresses. (**A**) Expression of five tissues of *M. coruscus* in different tissues in the natural state. (**B**) Expression of three tissues of thick-shelled mussels under an abiotic stress (heat stress). (**C**) Expression of three tissues of *M. coruscus* under a biotic stress (*V. lysogeneticus* stress).

Our investigation explored the expression patterns of HECT genes across various mussel tissues and developmental stages, utilizing a combination of published and unpublished RNA-seq datasets. Analysis of RNA-seq data from five distinct adult mussel tissues—blood, gill, mantle, gonad, and foot—facilitated the characterization of HECT expression profiles in *M. coruscus*. Notably, the analysis revealed that, on average, HECT genes exhibited elevated expression levels in the blood and gill, while displaying lower expression in the gonad and foot. Heat maps generated using FPKM values illustrated the tissue-specific expression patterns of HECT genes across various tissues (Figure 7A). Specifically, *Mco_HERC4* and *Mco_HECDT2* demonstrated robust expression primarily in the gill, while *Mco_UPL1* and *Mco_HERC1* showcased elevated levels in the blood. Furthermore, heightened expression was observed for *Mco_HERC2* and *Mco_UPL2* in the mantle. However, it is noteworthy that most HECT genes displayed expression in at least one tissue, suggesting their involvement across diverse physiological domains.

To investigate the expression dynamics of HECT genes in *M. coruscus* under abiotic and biotic stresses, we analyzed RNA-seq datasets from different tissues (adductor muscle, gill, and mantle) subjected to heat and *V. alginolyticus* stress. Following heat stress, four genes—*Mco_UPL3*, *Mco_HUWE1*, *Mco_NEDD4*, and *Mco_UBE3C*—exhibited upregulation in the gill, while only *Mco_HUWE1* and *Mco_UPL3* showed upregulation in the mantle and adductor tissues, respectively (Figure 7B). Remarkably, under *V. alginolyticus* stress, HECT genes were prominently expressed in gill tissues. However, only *Mco_UPL3* displayed significant upregulation, while the expression of *Mco_HECTD3*, *Mco_UBE3A*, *Mco_HERC1*, *Mco_HACE1*, *Mco_AREL1*, *Mco_EDD1*, and *Mco_TRIP12* was significantly downregulated. Additionally, *Mco_HECW1* exhibited notable upregulation, and *Mco_HECDT2* and *Mco_UPL2* showed significant downregulation in the mantle. Conversely, the expression in the adductor muscle remained largely unaltered (Figure 7C).

### 3.8. Gene Ontology Analysis of the HECT Gene

**Figure 8:**
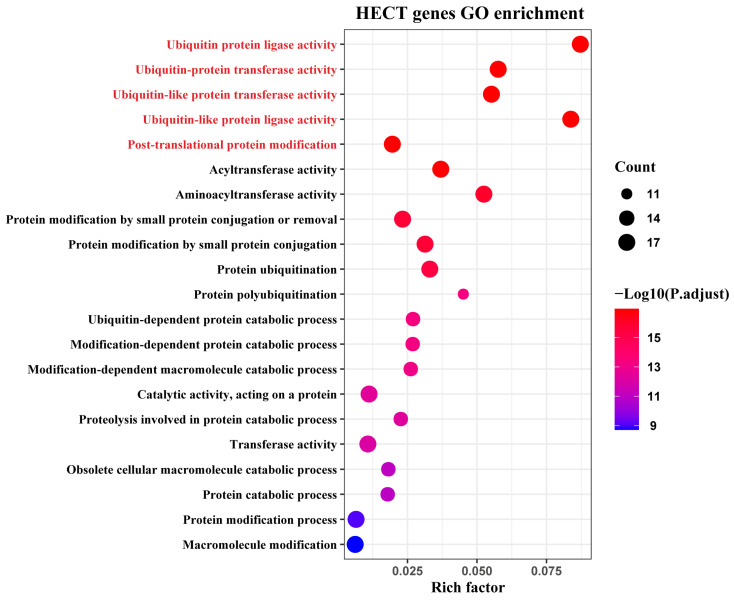
Gene ontology (GO) annotation results for HECT genes. Different functional categories are represented by different colors.

To further investigate the functions and metabolic pathways of the identified HECT E3 ligases in *M. coruscus*, we conducted a gene ontology (GO) enrichment analysis. The results indicated that 22 genes were significantly enriched in 51 GO terms (*p*-adjust < 0.05). These genes were predominantly associated with protein modification and degradation mechanisms, with a particular focus on pathways involving ubiquitination and ubiquitin-like modifications. The five most enriched GO terms were ubiquitin protein ligase activity (GO:0061630), ubiquitin-like protein transferase activity (GO:0019787), ubiquitin-like protein ligase activity (GO:0061659), acyltransferase activity (GO:0016746), and ubiquitin-protein transferase activity (GO:0004842) (Figure 8 and Table S4).

## 4. Discussion

Gene duplication is a pivotal factor in the evolution of gene families, contributing to the enhancement of gene structure and functional diversity through the generation of new family members [61,62]. In this investigation, we identified 22 HECT genes in the genome of *M. coruscus*, distributed across 11 chromosomes. Comparative analysis with other bivalves such as *M. galloprovincialis*, *M. yessoensis*, *C. gigas*, and *R. philippinarum* revealed a higher number of HECTs in *M. coruscus*, suggesting diverse functional roles for HECT genes in this species. While tandem duplications and segmental duplications are common types of gene duplications [63], our study did not uncover a substantial presence of these duplications in the *M. coruscus* HECT gene family. Notably, the *HECW* HECT gene originated from tandem duplication, and the *Mco_UPL1*, *Mco_UPL2*, and *Mco_UPL3* genes stemmed from segmental duplication, while the remainder originated from dispersed duplication. The observation that HECT genes in *M. coruscus* exhibit a high number of introns may indicate conservation, akin to early eukaryotes possessing genes with abundant introns [64]. Phylogenetic analysis of HECT genes in bivalves (*M. galloprovincialis*, *M. yessoensis*, *R. philippinarum*), and model organisms (*H. sapiens*, *D. melanogaster*, *D. rerio*, and *Drosophila*), along with the corresponding HECT homology classification, revealed six major categories (Figure 1) [61]. This classification aligns with the HECT classification observed in *R. philippinarum* [23], underscoring the relative evolutionary conservatism of the HECT E3 gene family in *M. coruscus*. Based on the phylogenetic tree, the NEDD4 subfamily and UPL subfamily HECT genes were most abundant among bivalves, featuring five and three genes, respectively, in *M. coruscus*. Conversely, *R. philippinarum* exhibited the fewest NEDD4 subfamily HECT genes, with only one identified among the bivalve sequences. Additionally, we noted that Class II harbors the most numerous and diverse types of HECT genes, known as key regulators of membrane proteins [65,66]. Numerous studies have demonstrated the crucial role of genes in this family in balancing tolerance and immunity [66]. Consideration of sequence, structure, and functional relationships suggests potential interactions with proteins and ligands.

Intertidal shellfish are subjected to a diverse array of environmental factors, with gills playing a pivotal role in facilitating gas exchange [67]. They facilitate oxygen uptake during submersion and prevent dehydration upon exposure to air. Beyond their respiratory function, the gills of shellfish serve as crucial organs for filtration and ingestion, essential for survival and adaptation to aquatic habitats. In response to environmental stressors such as temperature fluctuations and high salinity, gills undergo changes that impact respiratory efficiency, osmoregulation, and immune defense mechanisms [68]. Studies suggest that these adaptive changes in gill function enable shellfish to effectively manage stress, although chronic or severe stress can adversely affect physiological processes and overall survival [69,70,71]. Furthermore, in bivalves, blood plays an equally crucial role in adaptation to the intertidal environment. This blood transports nutrients, gases, and immune cells, contributing to osmoregulation, maintaining internal stability during tidal fluctuations, and preventing salinity changes [72]. Research on clams, oysters, and mussels underscores the significant role of hemocytes, immune cells in shellfish blood, in responding to environmental stressors, particularly pathogen exposure [73,74,75]. In the present investigation, *M. coruscus* HECT genes exhibited common expression in the examined tissues, notably in gills and blood. The specific high expression of HECT genes in the hemolymph and gill tissues of *M. coruscus* implies their potential involvement in regulating homeostatic tolerance and immune response in these organisms.

After being passed from ubiquitin-activating enzymes (E1) to ubiquitin-conjugating enzymes (E2), ubiquitin ligases co-ubiquitinate proteins (E3). The ubiquitin–proteasome system is one of the many intricate processes of ubiquitin-mediated protein degradation that is highly selective and degrades a significant amount of proteins in cells [76]. Abiotic stresses can induce the accumulation of misfolded or unfolded proteins, thereby triggering the generation of reactive oxygen species (ROS) [77]. Extensive evidence has demonstrated that intertidal shellfish endure high-temperature stress during the summer, a circumstance that may induce protein misfolding or denaturation, resulting in protein aggregation and the loss of physiological function. The E3 ligase ubiquitin is recognized as a pivotal factor in the proteasome pathway (UPP) [78], participating in the repair of damaged proteins under abiotic stresses [79,80]. We observed a significant upregulation of several HECT genes in the gill tissues of *M. coruscus* under heat stress conditions, including *Mco_UPL3*, *Mco_HUWE1*, *Mco_NEDD4*, and *Mco_UBE3C*. It has been proposed that *UPL3* is involved in protein ubiquitination, although studies on its exact physiological role in animals are more limited [81,82]. *HUWE1* is a key member of the E3 ligase family with a eukaryotic HECT structural domain. *HUWE1* regulates the turnover of various substrates, including MCL-1, P53, and c-MYC, thus participating in a broad spectrum of biological processes, including but not limited to apoptosis, autophagy, proliferation, differentiation, DNA damage repair, and stress responses [83,84,85]. NEDD4 primarily participates in the regulation of ubiquitin-mediated protein degradation, contributing to various cellular pathways that impact growth, development, and immunity [86]. UBE3C is associated with HECT and is involved in protein degradation pathways that affect the cell cycle and DNA repair in animals [87].

In terms of biotic stress, *Vibrio* vulnificus emerges as a significant pathogen in cultivated shellfish, causing a mortality rate of up to 90–100% within 24 h in infected bivalves, with associated industrial losses typically reaching 60% [88,89]. In our study, the expression of the HECT gene family in gill tissues decreased following stress induced by *V. alginolyticus*. This observation suggests a potential impact of these genes on the regulation of immune responses in *M. coruscus*. A prior investigation by YI et al. revealed a significant downregulation of *HUWE1* and *TRAF6* expression during white spot syndrome virus (WSSV) infection. This downregulation disrupted the ubiquitination of p53, resulting in apoptosis and reactive oxygen species (ROS) signaling through the accumulation of p53. Ultimately, this cascade inhibited viral invasion in mud crabs, offering a novel molecular mechanism for invertebrates to resist viral infections [90]. Our study detected an upregulation of *Mco_UPL3* under both heat and Vibrio stress, highlighting their pivotal roles in cellular processes such as protein ubiquitination and degradation. The functions of the UPL3 gene, encompassing mediation of protein stability, cellular signaling, and immune regulation, suggest a crucial role in regulating the thermoimmune response, particularly post-stress heat exposure. The diminished expression of HECT genes in mud crabs was found to be correlated with a reduction in UPL3 gene expression. The interaction between the diminished expression of HECT genes and the selective upregulation of UPL3 genes potentially orchestrated an adaptive immune response, enhancing resistance to stress. In conclusion, genes associated with the HECT structural domain may possess a regulatory role in the thermal immune response in bivalves. Nevertheless, further studies are imperative to comprehensively elucidate the specific functions of these genes and their roles in both biotic and abiotic stresses.

## 5. Conclusions

In this investigation, we characterized the HECT genes within the genome of *M. coruscus*, elucidating their molecular features and phylogenetic relationships. A total of 22 HECT genes were identified and categorized into six major types, all containing conserved HECT structural domains. Furthermore, we examined the expression patterns of HECT genes in *M. coruscus* exposed to both abiotic (high-temperature) and biotic (*V. alginolyticus*) stresses and performed functional annotation and enrichment analysis of these genes in the mussels. The findings revealed that the identified HECT genes in *M. coruscus* were enriched for molecular functions, including ubiquitination, degradation, and protein modification. These genes were significantly expressed in gill and blood tissues, with some showing marked upregulation in response to high-temperature and Vibrio vulnificus stress. This suggests potential roles for HECT genes in essential functions such as protein degradation and immunomodulation in mussels. Our results provide useful information for breeding for resilience in *M. coruscus*.

## Figures and Tables

**Table 1:** Summary of twenty-two HECT genes identified in *M. coruscus*.

No	Gene Name	Gene ID	CDSa Length	Protein Length	HECT Domain	Molecular Weight	Theoretical	Chromosome	Location
			(bp)	(aa)	Location (aa)	(kDa)	pI		
1	*Mco_NEDD4*	Maker00024065	1545	514	264–510	59,629.23	8.25	chr8	11,448,585–11,509,784
2	*Mco_EDD1*	Maker00026964	6696	2231	1935–2228	245,120.18	5.43	chr12	18,904,970–19,014,437
3	*Mco_UPL3*	Maker00035072	1608	535	450–532	60,513.88	4.62	chr5	45,075,511–45,107,379
4	*Mco_HERC4*	Maker00002539	384	127	11–106	15,283.79	5.63	chr10	81,621,876–81,717,176
5	*Mco_HERC1*	Maker00017428	9186	3061	2936–3022	338,818.36	5.84	chr8	30,444,670–30,540,646
6	*Mco_AREL1*	Maker00023631	1536	511	183–509	58,122.03	5.58	chr3	31,450,908–31,484,837
7	*Mco_UBE3A*	Maker00027142	2118	705	556–675	79,286.27	5.69	chr4	93,795,246–93,823,387
8	*Mco_HACE1*	Maker00030301	1266	421	194–414	47,852.62	5.63	chr9	115,378,105–115,430,703
9	*Mco_SMURF2*	Maker00031130	2328	775	516–772	86,570.70	6.24	chr10	2,742,196–2,791,928
10	*Mco_UPL1*	Maker00034862	1332	443	350–428	50,020.41	4.6	chr1	56,026,608–56,033,160
11	*Mco_HUWE1*	Maker00035590	11,616	3871	3613–3868	427,315.87	5.16	chr1	109,925,874–110,051,336
12	*Mco_UPL2*	Maker00001812	2172	723	578–653	82,200.81	5.26	chr6	99,678,904–99,683,847
13	*Mco_UBE3C*	Maker00001502	1821	606	350–604	69,595.05	5.36	chr1	53,415,654–53,447,223
14	*Mco_HECTD1*	Maker00005002	2991	996	487–994	112,627.93	5.02	chr3	99,488,382–99,510,834
15	*Mco_HECDT2*	Maker00005191	1056	351	139–348	39,778.40	9.29	chr8	111,081,717–11,114,3368
16	*Mco_TRIP12*	Maker00005211	4692	1563	1254–1562	171,572.52	8.14	chr3	135,995,715–136,168,882
17	*Mco_HECW1*	Maker00007292	1494	497	208–491	58,686.15	8.3	chr2	109,062,131–109,097,211
18	*Mco_HECW2*	Maker00007303	4050	1349	992–1347	151,476.02	6.19	chr2	108,953,278–108,963,278
19	*Mco_UBE3B*	Maker00008133	1485	494	144–492	56,659.97	6.49	chr7	102,429,555–102,469,658
20	*Mco_HECTD3*	Maker00011666	2274	757	511–715	87,274.83	5.54	chr1	5,886,440–5,977,704
21	*Mco_WWP1*	Maker00013008	1371	456	137–454	54,119.19	6.37	chr5	125,313,868–125,339,147
22	*Mco_HERC2*	Maker00015302	11,241	3746	3603–3722	412,033.61	5.92	chr4	33,350,025–33,456,457

**Table 2:** The E3 ubiquitin ligase-like subfamily of the HECT structural domain of *M. coruscus*.

HECT Subfamily	Gene Name	Acc. No.	Presence in Bivalvia Species *
NEDD4	*Mco_NEDD4*	Maker00024065	*M. coruscus(5)*, *M. galloprovincialis(4)*, *C. gigas(4)*, *R. philippinarum(1)*, *M. yessoensis(4)*
	*Mco_SMURF2*	Maker00031130	
	*Mco_WWP1*	Maker00013008	
	*Mco_HECW1*	Maker00007292	
	*Mco_HECW2*	Maker00007303	
UPL1-3	*Mco_UPL1*	Maker00035072	*M. coruscus(3)*, *M. galloprovincialis(0)*, *C. gigas(0)*, *R. philippinarum(2)*, *M. yessoensis(0)*
	*Mco_UPL2*	Maker00034862	
	*Mco_UPL3*	Maker00001812	
HACE1	*Mco_HACE1*	Maker00030301	*M. coruscus(1)*, *M. galloprovincialis(1)*, *C. gigas(1)*, *R. philippinarum(1)*, *M. yessoensis(1)*
HUWE1	*Mco_HUWE1*	Maker00035590	*M. coruscus(1)*, *M. galloprovincialis(1)*, *C. gigas(1)*, *R. philippinarum(1)*, *M. yessoensis(1)*
KIAA0317	*Mco_AREL1*	Maker00023631	*M. coruscus(1)*, *M. galloprovincialis(1)*, *C. gigas(1)*, *R. philippinarum(1)*, *M. yessoensis(1)*
UBE3A/E6-AP	*Mco_UBE3A*	Maker00027142	*M. coruscus(1)*, *M. galloprovincialis(1)*, *C. gigas(1)*, *R. philippinarum(1)*, *M. yessoensis(1)*
SMALL HERCs	*Mco_HERC4*	Maker00002539	*M. coruscus(1)*, *M. galloprovincialis(1)*, *C. gigas(1)*, *R. philippinarum(1)*, *M. yessoensis(1)*
UBE3B/3C	*Mco_UBE3B*	Maker00008133	*M. coruscus(2)*, *M. galloprovincialis(1)*, *C. gigas(2)*, *R. philippinarum(1)*, *M. yessoensis(2)*
	*Mco_UBE3C*	Maker00001502	
TRIP12	*Mco_TRIP12*	Maker00005211	*M. coruscus(1)*, *M. galloprovincialis(0)*, *C. gigas(1)*, *R. philippinarum(1)*, *M. yessoensis(1)*
HECTD1	*Mco_HECTD1*	Maker00005002	*M. coruscus(1)*, *M. galloprovincialis(1)*, *C. gigas(1)*, *R. philippinarum(1)*, *M. yessoensis(1)*
HECTD2	*Mco_HECDT2*	Maker00005191	*M. coruscus(1)*, *M. galloprovincialis(1)*, *C. gigas(1)*, *R. philippinarum(0)*, *M. yessoensis(1)*
HECTD3	*Mco_HECTD3*	Maker00011666	*M. coruscus(1)*, *M. galloprovincialis(1)*, *C. gigas(1)*, *R. philippinarum(1)*, *M. yessoensis(1)*
EDD/UBR5	*Mco_EDD1*	Maker00026964	*M. coruscus(1)*, *M. galloprovincialis(1)*, *C. gigas(1)*, *R. philippinarum(1)*, *M. yessoensis(1)*
G2E3	Not found	Not found	*M. coruscus(0)*, *M. galloprovincialis(0)*, *C. gigas(1)*, *R. philippinarum(0)*, *M. yessoensis(1)*
LARGE HERCs	*Mco_HERC1*	Maker00017428	*M. coruscus(1)*, *M. galloprovincialis(1)*, *C. gigas(1)*, *R. philippinarum(1)*, *M. yessoensis(1)*
LARGE HERCs	*Mco_HERC2*	Maker00015302	*M. coruscus(1)*, *M. galloprovincialis(0)*, *C. gigas(1)*, *R. philippinarum(1)*, *M. yessoensis(1)*
Not in any subfamily			*M. coruscus(0)*, *M. galloprovincialis(1)*, *C. gigas(1)*, *R. philippinarum(1)*, *M. yessoensis(1)*

* Only the genomes specifically mentioned for use in this study were selected; not all bivalve genomes were included.

**Table 3:** Secondary structure prediction and subcellular localization prediction of HECT proteins in *M. coruscus*.

Proteins	Alpha	Beta	Random	Extended	Subcellular
	Helix	Turn	Coil	Stand	Location Prediction
*Mco_NEDD4*	34.82%	6.03%	48.83%	10.31%	Nucleus
*Mco_EDD1*	34.02%	6.28%	42.94%	16.76%	Cytoplasm
*Mco_UPL3*	50.47%	5.05%	35.70%	8.79%	Nucleus
*Mco_HERC4*	53.54%	5.51%	25.98%	14.96%	Cytoplasm
*Mco_HERC1*	36.92%	8.95%	35.41%	18.72%	Nucleus
*Mco_AREL1*	40.90%	4.89%	34.44%	19.77%	Cytoplasm
*Mco_UBE3A*	42.55%	4.82%	40.43%	12.20%	Cytoplasm
*Mco_HACE1*	44.66%	4.51%	35.87%	14.96%	Cytoplasm
*Mco_SMURF2*	19.23%	8.26%	51.35%	21.16%	Nucleus
*Mco_UPL1*	38.83%	6.77%	42.44%	11.96%	Nucleus
*Mco_HUWE1*	44.33%	4.55%	40.02%	11.11%	Nucleus
*Mco_UPL2*	41.63%	4.98%	40.39%	13.00%	Plasma membrane
*Mco_UBE3C*	48.68%	7.10%	28.88%	15.35%	Plasma membrane
*Mco_HECTD1*	36.55%	4.62%	44.08%	14.76%	Cytoplasm
*Mco_HECDT2*	40.17%	6.55%	35.90%	17.38%	Cytoplasm
*Mco_TRIP12*	39.60%	7.49%	38.20%	14.72%	Nucleus
*Mco_HECW1*	50.30%	5.43%	33.60%	10.66%	Mitochondria
*Mco_HECW2*	28.54%	5.86%	51.22%	14.38%	Nucleus
*Mco_UBE3B*	48.38%	5.26%	33.00%	13.36%	Cytoplasm
*Mco_HECTD3*	35.40%	4.10%	43.73%	16.78%	Nucleus
*Mco_WWP1*	37.28%	6.80%	41.01%	14.91%	Cytoplasm
*Mco_HERC2*	38.49%	6.19%	38.60%	16.71%	Nucleus

## Data Availability

The original contributions presented in the study are included in the article/Supplementary Material, further inquiries can be directed to the corresponding author.

## Supplementary Materials

The following supporting information can be downloaded at: https://www.mdpi.com/article/10.3390/genes15081085/s1 [18,50,91,92,93], Table S1: Statistics of all genome project numbers; Table S2: A total of 148 HECT sequences in 10 species; Table S3: The BioProject number of RNA-seq datasets; Table S4: Gene ontology (GO) annotation results for the HECT gene of *M. coruscus*.
