# Supplementary material for: Genome-Wide Identification, Characterization, and Transcriptional Profile of the HECT E3 Ubiquitin Ligase Gene Family in the Hard-Shelled Mussel Mytilus coruscus Gould
Source: Genes (Basel). 2024 Aug 16;15(8):1085. doi: 10.3390/genes15081085 (PMC11353290; doi:10.3390/genes15081085)
Supplement: Supplementary file 1 [file genes-15-01085-s001.zip › Supplementary Information/Table S1.docx]

**Table S1** Statistics of all genome project numbers

| Species | BioProject number | DOI | Reference |
| --- | --- | --- | --- |
| *Mytilus galloprovincialis* | NA | Unpublish data |  |
| *Mytilus coruscus* | NA | Unpublish data |  |
| *Ruditapes philippinarum* | PRJNA479743 | 10.1016/j.cbd.2023.101060. | (Yan et al., 2019) |
| *Patinopecten yessoensis* | MolluscDB | 10.1093/nar/gkaa918 | (Liu et al., 2021) |
| *Crassostrea gigas* | PRJEB35351 | 10.1093/gigascience/giab020. | (Peñaloza et al., 2021) |

**Reference**

Liu, F., Li, Y., Yu, H., Zhang, L., Hu, J., Bao, Z. and Wang, S., 2021. MolluscDB: an integrated functional and evolutionary genomics database for the hyper-diverse animal phylum Mollusca. Nucleic Acids Research 49, D988-D997.

Peñaloza, C., Gutierrez, A.P., Eöry, L., Wang, S., Guo, X., Archibald, A.L., Bean, T.P. and Houston, R.D., 2021. A chromosome-level genome assembly for the Pacific oyster *Crassostrea gigas*. Gigascience 10.

Yan, X., Nie, H., Huo, Z., Ding, J., Li, Z., Yan, L., Jiang, L., Mu, Z., Wang, H., Meng, X., Chen, P., Zhou, M., Rbbani, M.G., Liu, G. and Li, D., 2019. Clam Genome Sequence Clarifies the Molecular Basis of Its Benthic Adaptation and Extraordinary Shell Color Diversity. iScience 19, 1225-1237.
