# Supplementary material for: Genome-Wide Identification, Characterization, and Transcriptional Profile of the HECT E3 Ubiquitin Ligase Gene Family in the Hard-Shelled Mussel Mytilus coruscus Gould
Source: Genes (Basel). 2024 Aug 16;15(8):1085. doi: 10.3390/genes15081085 (PMC11353290; doi:10.3390/genes15081085)
Supplement: Supplementary file 1 [file genes-15-01085-s001.zip › Supplementary Information/Table S3.docx]

**Table S3** The BioProject number of RNA-seq datasets

| Type | BioProject number | DOI | Reference |
| --- | --- | --- | --- |
| Tissue-specific | NA | Unpublish data |  |
|  |  |  |  |
| Heat stress | PRJNA793930 | 10.1016/j.cbd.2023.101060. | (Wang et al., 2023) |
|  |  |  |  |
| Vibrio stress | PRJNA978366 | 10.1016/j.fsi.2023.109301. | (Li et al., 2024) |

**Reference**

Li, H., Zhao, J., Li, Y., Dong, Z., Lin, S., Guo, B. and Qi, P., 2024. Transcriptome analysis reveals tissue-specific responses of Mytilus unguiculatus to Vibrio alginolyticus infection. Fish Shellfish Immunol 144, 109301.

Wang, Y.X., Lin, S.R., Xu, L.Z., Ye, Y.Y., Qi, P.Z., Wang, W.F., Buttino, I., Li, H.F. and Guo, B.Y., 2023. Comparative transcriptomic analysis revealed changes in multiple signaling pathways involved in protein degradation in the digestive gland of Mytilus coruscus during high-temperatures. Comp Biochem Physiol Part D Genomics Proteomics 46, 101060.
